# Mannitol Preserves the Viscoelastic Properties of Hyaluronic Acid in an In Vitro Model of Oxidative Stress

**DOI:** 10.1007/s40744-014-0001-8

**Published:** 2014-07-25

**Authors:** Thierry Conrozier, Pierre Mathieu, Marguerite Rinaudo

**Affiliations:** 1 Service de Rhumatologie, Centre Hospitalier de Belfort-Montbéliard, Belfort, France; 2 Rhumatologue, Chazelles-sur-Lyon, France; 3 Biomaterials Applications, Grenoble, France

**Keywords:** Antioxidant, Hyaluronic acid, Hydrogen peroxide, Mannitol, Osteoarthritis, Reactive oxygen species, Rheology, Viscoelastic properties, Viscosupplementation

## Abstract

**Introduction:**

Viscosupplementation by intra-articular injection of hyaluronic acid (HA) is a widely used treatment for lower limb osteoarthritis. However, the injected HA is rapidly degraded by reactive oxygen species (ROS), limiting its time of intra-articular residence. Optimizing clinical effectiveness of viscosupplementation by reducing HA degradation in situ, and therefore increasing the time of contact with the diseased tissue, is a challenging research approach. Mannitol, a powerful ROS scavenger, is a good candidate for this. The aim of this study was to compare in vitro the resistance to ROS-mediated degradation of two marketed viscosupplements (one linear and one cross-linked) to that of two novel viscosupplements combining HA and mannitol.

**Methods:**

A HA viscosupplement at a concentration of 10 g/L (HA 1%), was compared to a HANOX-M, a novel viscosupplement made of a mixture of HA and mannitol. In a second experiment, Hylan G-F 20, a partially cross-linked viscosupplement, was compared to a HANOX-M-XL, a novel cross-linked viscosupplement made of a HA and mannitol (35 g/L). The four HA viscosupplements were subjected to oxidative stress generated by the addition of hydrogen peroxide (H_2_O_2_) and their rheological behavior (elastic moduli [G′], viscous moduli [G″], and complex viscosity [|η*|]) were compared before and after the oxidative stress exposure.

**Results:**

The two viscosupplements not containing mannitol HA were rapidly degraded by H_2_O_2_, as demonstrated by the dramatic decrease of |η*|. On the other hand, the rheological properties of HA containing mannitol were not substantially modified in the presence of H_2_O_2_.

**Conclusion:**

This in vitro study demonstrates that mixing mannitol with HA protects the viscosupplement from ROS-mediated degradation and might therefore increase its intra-articular residence time without substantially modifying its rheological behavior. This in vitro study has to be followed by clinical trials designed to assess whether the addition of mannitol to HA might improve the efficiency and/or the duration of action of viscosupplementation.

**Electronic supplementary material:**

The online version of this article (doi:10.1007/s40744-014-0001-8) contains supplementary material, which is available to authorized users.

## Introduction

The hallmark of osteoarthritis (OA) is an increased degradation of the articular cartilage extracellular matrix (ECM) molecules, leading to progressive cartilage destruction, combined with several other joint tissue changes including bone remodeling (osteophyte formation, subchondral sclerosis, cyst formation), synovium low-grade inflammation, and frequently synovial fluid (SF) effusion [1].

Viscosupplementation by intra-articular injections of high molecular weight (HMW) hyaluronic acid (HA) is a widely used treatment for knee OA, aimed to restore the rheological homeostasis of the SF and to precipitate a restoration of normal HA metabolism [2, 3]. HA biologic functions depend on interactions with specific binding proteins called hyaladherins that modify the conformation of HA such as the cell surface hyaluronan receptor (CD44), HA-mediated motility (RHAMM), and hyaluronan-binding proteins of the ECM. Indeed, SF is a viscoelastic system consisting in a complex of HA and proteins constituting a physical 3D-network [4–7]. HA is an anionic polyelectrolyte, constituted of alternating units of *N*-acetylglucosamine and d-glucuronic acid molecules. In healthy SF the polysaccharide chain can be up to 12,000 disaccharide units and has an average MW of 4–6 million Da. HA is produced in synovial joints by hyalocytes located in the synovial membrane and is released into the SF [3]. It plays a major role in lubrication, shock absorption, and viscoelastic behavior of the SF [3] involving entanglements in HA and proteins/HA association based mainly on electrostatic interactions. This viscoelastic behavior, which is directly related to both MW and concentration of HA [8], allows the HA 3D network to adapt to the mechanical stress applied. At low shear, such as occurs during a slow movement, the linear chains of HA align slowly in the direction of flow and behave like a viscous fluid. When the joint is subjected to fast impact (i.e., running or jumping) HA molecules do not have time enough to realign and exhibit elastic behavior, allowing shock absorption. This change in behavior is called viscoelastic behavior based on the temporary network formed by chain entanglements (as observed in dynamic experiments) and usually corresponds to a non-Newtonian behavior in flow experiments.

In OA joints, SF viscoelasticity, and consequently ability to protect cartilage, are dramatically lowered when compared with healthy SF as a result of a decrease of both HA MW and concentration [2].

Viscosupplementation effect is not fully clarified and is probably due to several mechanisms such as promotion of endogenous HMW HA production [9], interaction with pain receptors, and various anti-inflammatory effects [10–16].

There are now more than 25 commercial viscosupplement formulations available worldwide from different manufacturers. These products widely vary in their MW, concentration, volume, indication, residence time into the joint [17–19], and recommended dosing regimens, ranging from one to five injections at weekly intervals. Despite the long history of this therapy and its inclusion in several guidelines for the management of OA subject to certain conditions [20–26], the use of viscosupplementation in OA remains a topic for debate regarding treatment efficacy and safety, ideal dosing regimen, cost-efficacy, differences between commercial preparations, and predictors of clinical response [27–30]. These discrepancies may originate from differences in efficacy between the studied products, varying in concentration, MW, and molecular cross-linkage [20]. Indeed, the HA injected into the joint is rapidly degraded, limiting the time of intra-articular residence from a few days for linear molecules [17] to a few weeks for the solutions of cross-linked HA [18, 19]. Among the many pathogenic mechanisms contributing to HA degradation, reactive oxygen species (ROS) derivatives play a major role [31, 32]. Then OA is a degenerative joint disease of multifactorial origin in the pathogenesis of which ROS play a deleterious effect [33]. It has been shown that interleukin-1β (IL-1β) activates the production by the chondrocytes of large amounts of ROS which are themselves involved in the production and/or the activation of collagenase matrix metalloproteinase-1 and in the phenomena of chondrocyte apoptosis [34]. In addition to their effect on the degradation of the ECM, ROS are directly involved in the mechanisms of degradation of HA. Optimizing clinical effectiveness of viscosupplementation by reducing HA degradation in situ, and therefore increasing the time of contact with the diseased tissue [35], is a challenging research approach. We [36] and others [32] have previously shown that the addition of mannitol (a polyol well known for its ROS-scavenging properties) with HA, protects the latter to ROS-mediated degradation and therefore may increase the injected HA residence time into the joint.

The aim of the present study was to compare in vitro the resistance to ROS-mediated-degradation of two marketed viscosupplements (one linear and one cross-linked) to that of two novel viscosupplements combining HA and mannitol.

## Methods

This article does not contain any studies with human or animal subjects performed by any of the authors.

### Viscosupplements to be Assessed

In the first experiment, a HA viscosupplement with an average MW of 800 kDa at a concentration of 10 g/L [HA 1%] (Go-on^®^; Rottapharm SpA, Monza, Italy), was compared to a HANOX-M (HAppyVisc^®^; LABRHA, Lyon, France), a novel viscosupplement made of a mixture of HA (15.5 g/L, average MW 1 MDa) and mannitol (35 g/L). Both were of biofermentation origin and were not cross-linked.

In the second experiment, Hylan G-F 20, a partially cross-linked viscosupplement, obtained from cockscomb extraction, at a HA concentration of 8 g/L with a stated MW of 6 MDa (Synvisc^®^; Sanofi-Aventis, France) was compared to a HANOX-M-XL (HAppyCross^®^; LABRHA), a novel cross-linked viscosupplement also made of a mixture of a non-animal origin HA (16 g/L) and mannitol (35 g/L).

The average MW of each linear HA was determined using steric exclusion chromatography using a chromatograph (Waters^®^ Alliance^®^ GPC/V 2000; Waters Corporation, Milford, MA, USA) equipped with three detectors in line (26). The injected concentration was 2 g/L, with an injection volume of 100 µL using two columns in series (OHpak^®^ 805 and 806; Shodex, Munich, Germany). All samples were filtered through a membrane with pores of 0.2 µm (Cellulose Acetate Membrane Filter; Sartorius, Surrey, UK) prior to injection in order to retain any aggregates. The eluent used was 0.1 M of NaNO_3_ at an elution temperature of 30 °C and a flow rate of 0.5 mL/min.

### Induction of Oxidative Stress

Hydrogen peroxide (H_2_O_2_) application is a well-known method to degrade polysaccharides through a radical mechanism [37]. The 4 HA viscosupplements mentioned above were subjected to oxidative stress generated by the addition of H_2_O_2_ (30%; Carl Roth GmbH & Co. KG, Lauterbourg, Germany) at a final concentration of 2.7% and/or 5.4% (v/v). For that purpose, the commercial samples were added of H_2_O_2_ in a ratio 10:1 and stirred before being placed rapidly on the rheometer (delay time between addition of H_2_O_2_ and first measurement was 2 min).

### Rheological Assessment

The rheological properties were measured using a cone-plate rheometer (ARES-G2 Rheometer; TA Instruments, New Castle, DE, USA) at 25 °C on the different solutions as a function of the oxidative stress time at room temperature. The dynamic experiments were carried out in the region of linear viscoelasticity, where the G′ elastic and G″ viscous moduli are independent of the applied frequency. The dynamic moduli expressed in Pa (G′, the elastic contribution, and G″, the storage contribution) and the complex viscosity (|η*|) were taken at the angular frequency (*ω*) of 1 Hz.

## Results

The two viscosupplements not containing mannitol HA were rapidly degraded by hydrogen peroxide, as demonstrated by the dramatic decrease of the complex viscosity (|η*|). On the other hand, the rheological properties of the HA solutions containing 35 g/L of mannitol were not substantially modified in the presence of H_2_O_2_ over a period of 15 or 30 min. The resistance to oxidative stress was much better for the cross-linked (HANOX-M-XL) than for the non-cross-linked (HANOX-M) HA containing mannitol, the cross-linking being the only difference between them (same origin, and same HA and mannitol concentrations). Likewise, at baseline, HANOX-M was much more viscous than HA 1%, because of its higher MW (average MW 1.2 MDa versus 789 kDa) and concentration (15.5 g/L versus 10 g/L).

**Fig. 1:**
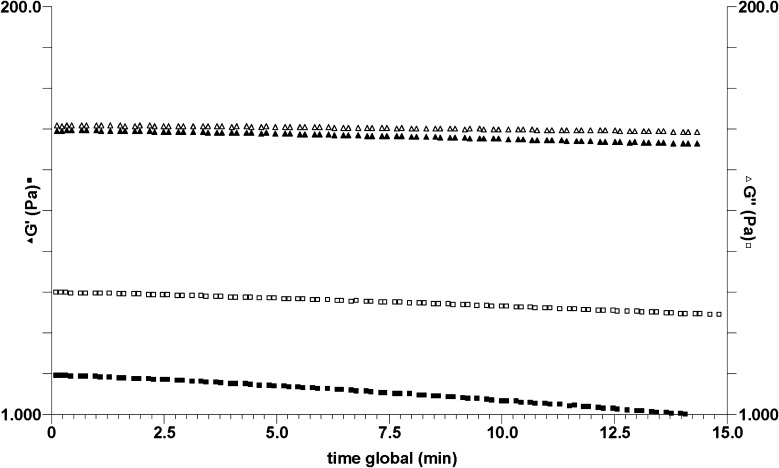
Elastic (G′) and storage (G″) moduli at 1 Hz of hyaluronic acid (HA) solutions as a function of oxidative degradation time on linear hyaluronans. *Filled triangle* G′ (Pa) *open triangle* G″ (Pa) on HANOX-M; *filled square* G′ (Pa) *open square* G″ (Pa) on HA 1%

**Fig. 2:**
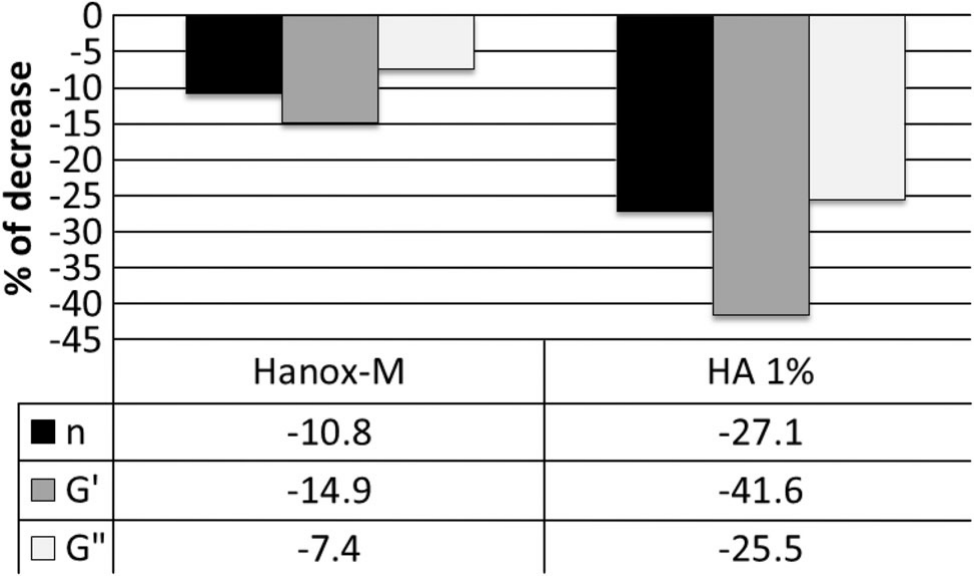
Decrease (%) of elastic (G′) and viscous (G″) moduli and complex viscosity (|η*|) after 15 min of oxidative stress by hydrogen peroxide. Comparison of two linear non cross-linked viscosupplements: HANOX-M and hyaluronic acid (HA) 1%

Figure 1 shows the rheological behaviors of the two linear viscosupplements. It clearly demonstrates that the presence of mannitol in HANOX-M stabilizes HA against oxidative degradation. Conversely, it shows that the elastic modulus (G′) of HA 1% solution was particularly decreased. The values of rheological parameters (elastic and viscous moduli, complex viscosity) taken after 15 min are represented in Fig. 2. This histogram is very indicative of the benefit of mannitol on HA stability after H_2_O_2_ exposure.

**Fig. 3:**
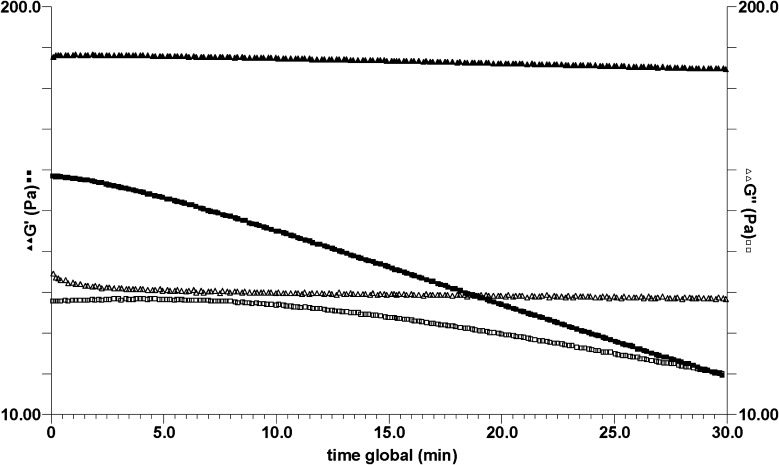
Elastic (G′) and storage (G″) moduli at 1 Hz of hyaluronic acid (HA) solutions as a function of oxidative degradation time on crosslinked hyaluronans. *Filled triangle* G′ (Pa) *open triangle* G′ (Pa) on HANOX-M-XL; *filled square* G′ (Pa) *open square* G″ (Pa) on Hylan G-F 20

**Fig. 4:**
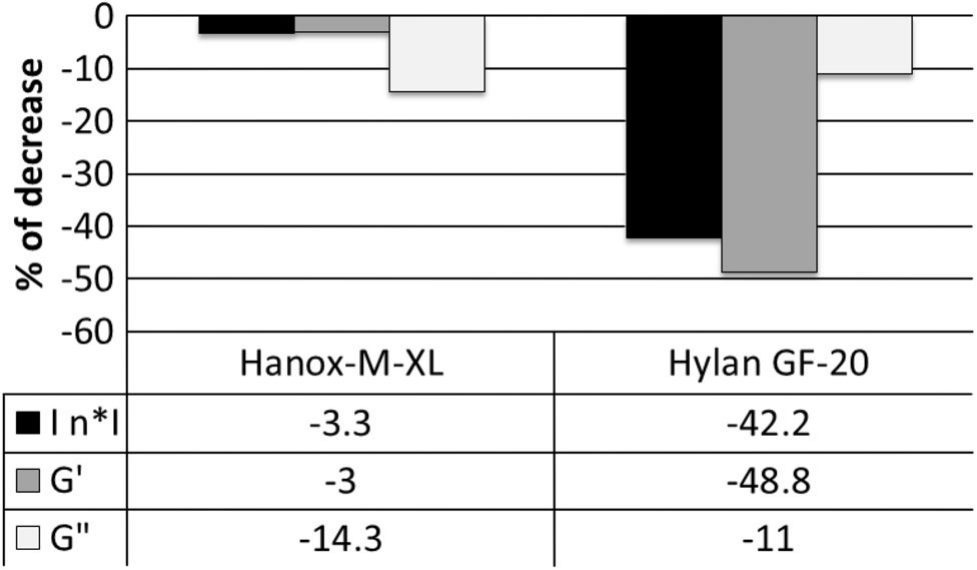
Decrease (%) of elastic (G′) and viscous (G″) moduli and complex viscosity (|η*|) after 15 min of oxidative stress by hydrogen peroxide. Comparison of two cross-linked viscosupplements: HANOX-M-XL and Hylan G-F 20

Under the same experimental conditions, the kinetics of degradation of the two cross-linked viscosupplements was compared (see Figs. 3, 4). The influence of mannitol is again obvious. Nevertheless, the level of the elastic modulus (G′) of HANOX-M-XL, which is much higher than that of Hylan G-F 20, indicates that its degree of cross-linkage is higher than that of Hylan G-F 20. The better resistance to degradation of HANOX-M-XL is likely due to both mannitol and its high level of cross-linkage. Figure 4 represents the rheological parameters after 15 min of oxidative degradation.

## Discussion

This in vitro study, using a powerful model of oxidative stress, shows that the addition of high concentration of mannitol (35 g/L) to HA protects HA from degradation. Mannitol (C_6_H_14_O_6_) is a polyol well known for its properties as a ROS scavenger. The beneficial effect related to its antioxidant power towards the rich reactive hydroxyl function has been shown in various diseases [38–41].

In viscosupplementation, the rationale of its combination with HA is legitimate: When administered intra-articularly, HA macromolecules of the viscosupplement, containing many OH groups, react with ROS, resulting in the rupture of the macromolecular chains and accelerated degradation by radical mechanism [31]. This rapid depolymerization of HA is the main cause for the short intra-articular half-life of viscosupplements made of non-cross-linked HA, cross-linking being another way to protect HA from degradation by ROS [18, 19]. The chemical characteristics of mannitol make it an antioxidant of choice in combination with HA. As with HA, mannitol has a very good safety profile, as demonstrated by animal tests showing that it was non-cytotoxic, non-genotoxic, non-carcinogenic, and non-mutagenic, even at high doses [42]. In humans, mannitol is widely used per os and by intravenous injection at very high concentrations, including hyperosmolar concentrations [43]. Its resistance to heat also permits sterilization by autoclaving, unlike other antioxidants that are thermolabile (e.g., polyphenols, vitamin C). Furthermore, mannitol does not increase the ionic strength of the medium and thus does not significantly alter the rheological behavior of the HA, and is hydrosoluble, unlike antioxidants such as vitamin E or betacarotene [44]. Two open-label clinical trials assessing viscosupplements containing antioxidants (mannitol 5 g/L [45] or its isomer sorbitol 40 g/L [46]) in patients suffering from knee OA have been recently published. In both, safety was similar to that of conventional viscosupplements not containing antioxidant, and none of them has revealed any serious or unexpected adverse effects. In the absence of a control group, the efficacy cannot be conclusively assessed; however, the published results strongly suggested the beneficial effects of the treatments on pain and function.

Our data also suggested that HA cross-linking, high MW and high concentration may play a role in the improved resistance to oxidative stress and are other effective ways to protect HA from degradation.

## Conclusion

In summary, this in vitro study demonstrates that mixing high concentrations of mannitol with HA, protects the viscosupplement from ROS-mediated degradation and might therefore increase the intra-articular residence time of the gel without substantially modifying its rheological behavior. Thereby, the combination of HA and mannitol might be a simple mean to improve the efficiency and/or the duration of action of joint viscosupplementation. These in vitro results fully justify the in vivo studies already in progress with both HANOX-M and HANOX-M-XL whose aims are to confirm or refute this exciting hypothesis and to assess the effectiveness and safety of this new promising association on large samples of patients.

## Electronic supplementary material

Below is the link to the electronic supplementary material.

Supplementary material 1 (PDF 187 kb)
