# Supplementary material for: Mannitol Preserves the Viscoelastic Properties of Hyaluronic Acid in an In Vitro Model of Oxidative Stress
Source: Rheumatol Ther. 2014 Jul 25;1(1):45–54. doi: 10.1007/s40744-014-0001-8 (PMC4883257; doi:10.1007/s40744-014-0001-8)
Supplement: Supplementary file 1 — Supplementary material 1 (PDF 187 kb) [file 40744_2014_1_MOESM1_ESM.pdf]

- Viscosupplementation by intra-articular injections of hyaluronic acid (HA) is a widely used treatment for knee osteoarthritis.
- However, HA injected into the joint is rapidly degraded by reactive oxygen species (ROS), limiting the time of intra-articular residence from a few days for linear molecules to a few weeks for the solutions of cross-linked HA, and therefore limiting its efficacy.
- Mannitol, through its ROS-scavenging properties, may avoid HA degradation by oxidative stress.
- HA viscosupplements with or without mannitol were subjected to oxidative stress (hydrogen peroxide) and their rheological behavior were assessed before and after the stress.
- The results clearly demonstrated that mannitol protects HA from ROS-mediated depolymerization and may be a simple means to improve HA duration of action and efficacy.

This summary slide represents the opinions of the authors. The study has been sponsored by LABRHA (Laboratoire de Rhumatologie Appliquée, 19 place Tolozan, 69001, Lyon, France). For a full list of acknowledgments and conflicts of interest for all authors of this article, please see the full text online. Copyright © The Author(s) 2014. Creative Commons Attribution Noncommercial License (CC BY-NC).
